# Prohibitins in neurodegeneration and mitochondrial homeostasis

**DOI:** 10.3389/fragi.2022.1043300

**Published:** 2022-11-03

**Authors:** Jesus Fernandez-Abascal, Marta Artal-Sanz

**Affiliations:** ^1^ Andalusian Centre for Developmental Biology (CABD), CSIC-Universidad Pablo de Olavide-Junta de Andalucía, Sevilla, Spain; ^2^ Department of Molecular Biology and Biochemical Engineering, Universidad Pablo de Olavide, Seville, Spain

**Keywords:** neurodegeneration, aging, nervous system, prohibitin (PHB), mitochondria

## Abstract

The incidence of age-related neurodegenerative disorders has risen with the increase of life expectancy. Unfortunately, the diagnosis of such disorders is in most cases only possible when the neurodegeneration status is already advanced, and symptoms are evident. Although age-related neurodegeneration is a common phenomenon in living animals, the cellular and molecular mechanisms behind remain poorly understood. Pathways leading to neurodegeneration usually diverge from a common starting point, mitochondrial stress, which can serve as a potential target for early diagnosis and treatments. Interestingly, the evolutionarily conserved mitochondrial prohibitin (PHB) complex is a key regulator of ageing and metabolism that has been associated with neurodegenerative diseases. However, its role in neurodegeneration is still not well characterized. The PHB complex shows protective or toxic effects in different genetic and physiological contexts, while mitochondrial and cellular stress promote both up and downregulation of PHB expression. With this review we aim to shed light into the complex world of PHB’s function in neurodegeneration by putting together the latest advances in neurodegeneration and mitochondrial homeostasis associated with PHB. A better understanding of the role of PHB in neurodegeneration will add knowledge to neuron deterioration during ageing and help to identify early molecular markers of mitochondrial stress. This review will deepen our understanding of age-related neurodegeneration and provide questions to be addressed, relevant to human health and to improve the life quality of the elderly.

## Introduction

Aging has expanded the prevalence of neurodegenerative diseases (ND), which have become one of the greatest challenges in public health and are the leading cause of disability in the world (GBD 2019 Ageing Collaborators, 2022). Since the diagnosis of most of ND only occurs when the nervous system is considerably damaged and the symptoms become evident, the study of the early processes of neurodegeneration will be an important focus of research in the next years, especially for the identification of new therapeutic targets and molecular markers of disease. Among the several ND, their etiology is usually classified based on their symptomatology and the area of the nervous system where the neurodegeneration occurs (Dugger and Dickson, 2017; Erkkinen et al., 2018). For example, loss of dopaminergic neurons in the substantia nigra and the presence of Lewy bodies cause Parkinson’s disease (PD), characterized by tremor and bradykinesia and progressive rigidity (Ye et al., 2022). Similarly, a progressive degeneration starting from the cortical area and spreading to other brain’s structures, including amygdala and hippocampus, and the presence of senile plaques are characteristic of Alzheimer’s disease (AD) (DeTure and Dickson, 2019). Moreover, loss of neurons in the cortex and striatum due to expansion of CAG repeats in the huntingtin gene causes chorea, dementia and psychiatric problems in Huntington’s disease (HD) (Hong et al., 2021). In amyotrophic lateral sclerosis (ALS), degeneration of motor neurons in cortex, brainstem and spinal cord cause muscle weakness and atrophy (Grad et al., 2017). Finally, neurodegeneration in temporal and frontal cortices develop in behavioral and locomotor problems that are associated with frontotemporal dementia (FTD) (Bang et al., 2015). However, the cause(s) of the neurodegeneration in the above and other CNS diseases may be very different but converge to mitochondria. Indeed, environmental, genetic and/or spontaneous alterations in mitochondrial homeostasis may cause mitochondrial stress, which consist in damage in DNA, proteins and/or lipids that trigger quality-control pathways to overcome the damage (Youle and van der Bliek, 2012). While ND and their subtypes have different etiologies, the beginning of the neurodegeneration process in most of them share pathways related with early mitochondrial stress (Lin and Beal, 2006).

Striking progress have been obtained in the past decades when it comes to elucidating mitochondrial function and its relationship with neurodegenerative diseases and aging. In PD, environmental exposure to 1-methyl-4-phenyl-1,2,3,6-tetrahydropyridine (MPTP) or pesticides cause mitochondrial disfunction, as well as genetic factors such as mutations in the PINK1, parkin, DJ-1, POLG, LRRK2, and SNCA genes, which are involved in mitochondrial pathways [reviewed in Borsche et al. (2021)]. In AD, mitochondrial disfunction also contributes to development of neurodegeneration, although is still not clear if an initial mitochondrial and bioenergetic alteration causes protein aggregation and further problems in the neuronal physiology, or whether upstream pathologies including aggregation trigger mitochondrial stress and progression of the disease [reviewed in Swerdlow (2018)]. In HD, despite its cause is purely genetic, the interactions of the mutated huntingtin protein with mitochondria is one of the earliest events in the development of the disease, and cause mitophagy, synaptic degeneration, defective mitochondrial transport, excessive mitochondrial fragmentation and failure to remove dead mitochondria [reviewed in Sawant et al. (2021)]. In ALS and FTD, the impairment of mitochondrial function has been widely studied, and many genes are involved in mitochondrial stress, dynamics, structure, bioenergetics and calcium buffering [reviewed in Smith et al. (2019) and Anoar et al. (2021)]. One of the major regulators of mitochondrial homeostasis is the prohibitin (PHB) complex (Lin and Beal, 2006; Artal-Sanz and Tavernarakis, 2009a). The PHB complex is formed by two prohibitin isoforms, prohibitin 1 (PHB1) and prohibitin 2 (PHB2), which form a heterodimeric ring-shaped complex in the mitochondrial inner membrane (Steglich et al., 1999; Nijtmans et al., 2000; Artal-Sanz et al., 2003). PHB-1 and PHB-2 subunits are interdependent for protein complex formation, leading the absence of one of them to the absence of the whole PHB complex (Berger and Yaffe, 1998; Nijtmans et al., 2000; Artal-Sanz et al., 2003). The PHB complex is strongly evolutionarily conserved among eukaryotes and is ubiquitously and abundantly expressed (Nijtmans et al., 2000; Coates et al., 2001). Prohibitins play important roles in many physiological events, including energy production, intracellular signaling, aging, metabolism, and apoptosis, however, their exact biochemical function remains to be clarified. Two predominant views have emerged for the function of the PHB complex; as a membrane-bound chaperone-like complex (Nijtmans et al., 2000; Nijtmans et al., 2002), and as a lipid scaffold-like complex (Merkwirth et al., 2008; Osman et al., 2009; Merkwirth et al., 2012). Although more work is needed to clarify its exact molecular function, evidences show a direct impact of the PHB complex on mitochondrial functionality. Indeed, imbalance in the cytoplasmic/mitochondrial ratio of protein levels by disruption of mitochondrial protein synthesis with thiamphenicol causes upregulation of prohibitin expression (Coates et al., 2001). This role in protein metabolism has been suggested to be carried out by the PHB complex binding directly to the newly synthetized products, avoiding their degradation and conferring stability (Nijtmans et al., 2002; Artal-Sanz and Tavernarakis, 2009a). On the contrary, lack of PHB complex induces the mitochondrial-specific unfolded protein response (UPR^mt^) (Hernando-Rodriguez and Artal-Sanz, 2018; Hernando-Rodriguez et al., 2018) and alters lipid metabolism, specially cholesterol synthesis and alters cardiolipin acylation (Merkwirth et al., 2008; Richter-Dennerlein et al., 2014; Hernando-Rodriguez and Artal-Sanz, 2018; Lourenco et al., 2021). Furthermore, deregulation of the PHB complex and mitochondrial dysfunction have been associated with many physiological processes like cancer [reviewed in Koushyar et al. (2015)], liver injuries [reviewed in Barbier-Torres and Lu (2020)], obesity and adipocyte-immune cell cross-talk in diabetes [reviewed in Ande et al. (2014) and in Mishra and Nyomba (2017)], degenerative disorders [reviewed in Signorile et al. (2019)], sex-based immune diseases [reviewed in Mishra and Nyomba (2019) and in Zi Xu et al. (2018)], ageing [reviewed in Thuaud et al. (2013)] and cell survival and apoptosis [reviewed in Peng et al. (2015)]. Importantly, in the recent years, PHB proteins have been proposed to have a role in several ND, including PD, AD, HD, ALS, FTD and others (Merkwirth et al., 2012; Belser and Walker, 2021). However, the cellular and molecular underpinnings of PHB function in the nervous system and in the context of ageing are still poorly understood. Here, we review the latest advances in neurodegeneration and mitochondrial homeostasis associated with PHB in the nervous system and propose important questions to be addressed in future research.

## Prohibitins in vertebrates

The study of PHB in the nervous system of vertebrates has been limited by the complexity of animal models and the short number of human samples. It has focused mainly on the correlation of molecular cues involved in mitochondrial homeostasis observed in cell cultures, and the study of protein expression and cellular viability under certain conditions.

### Mitochondrial function and complex assembly

**FIGURE 1:**
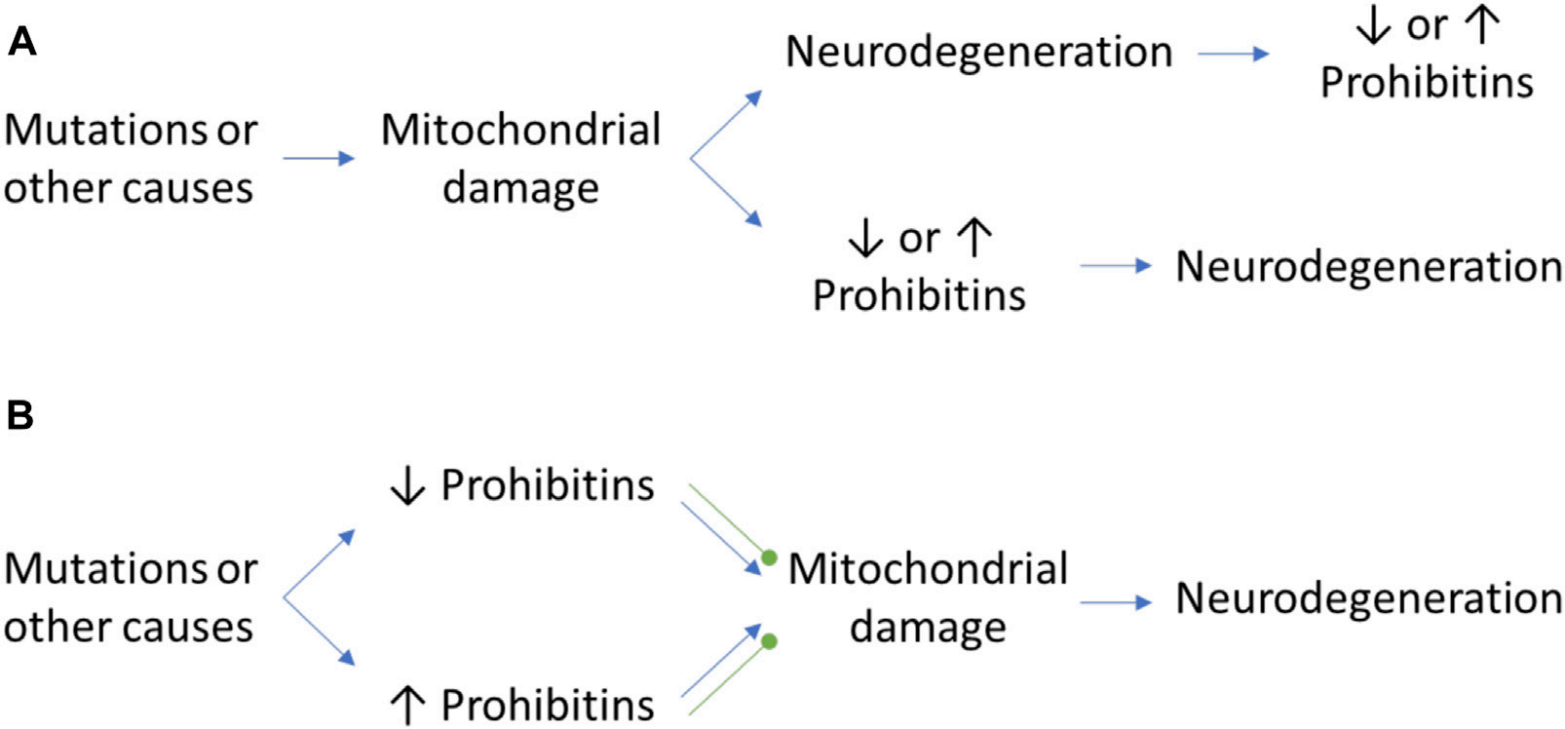
The controversial role of PHB in neurodegeneration. **(A)** Mitochondrial damage may cause neurodegeneration and a differential expression of PHB as a consequence of the neurodegenerative process or the activation of mitophagy pathways. Inversely, the mitochondrial damage itself may cause an alteration of PHB expression levels which may trigger the neurodegenerative process. **(B)** The alteration of PHB levels in normal conditions due to mutations in other proteins may have beneficial and detrimental effects that will cause the protection against mitochondrial damage or the promotion of it, respectively. Green round arrows: protection against mitochondrial damage, which prevents neurodegeneration.

One of the crucial aspects of neuronal survival is the correct assembly of mitochondrial complex I. Several cases of ALS and FTD are related with lower metabolic activity and energy production. For example, in a liquid chromatography tandem mass spectrometry study of ALS and FTD patients, Iridoy and colleagues observed that both PHB1 and PHB2 where downregulated, and Western blot (WB) analysis further confirmed a reduction of ≈15%–20% and ≈40%, respectively, in the spinal cord (Iridoy et al., 2018). On the other hand, haploinsufficiency of C9orf72, a protein closely related with ALS and FTD, causes a ≈50% degradation of the translocase TIMMDC1, and a ≈30% blockage of complex I assembly, as measured by two-dimensional BN/SDS–PAGE analysis (Wang et al., 2021a). Wang and colleagues demonstrated that in mice, the PHB complex is recruited by C9orf72 in the mitochondrial intermembrane space, which block the degradation of the translocase TIMMDC1 and facilitates complex I assembly (Wang et al., 2021a). Another protein involved in ALS and FTD, CHCHD10, have been related with PHB and motor neuron degeneration. In a recent study by Genin and colleagues, the authors observed in patient fibroblasts and mice by immunolabelling that in 48.1% of dying spinal motor neurons, PHB aggregates with Stomatin-Like Protein (SLP2) in the absence of CHCH10, which participate in the stability of the PHB complex and regulates motor neuron death (Genin et al., 2022). Since a normal level of PHB is required for the correct functioning of mitochondria, a decrease of PHB levels in specific tissues can be a consequence of reduced cell numbers due to the neurodegeneration process rather than an actual downregulation of PHB expression. Therefore, data should be analyzed with caution as further studies analyzing PHB expression levels are required to elucidate their role on the different stages of the neurodegenerative process and ageing (Figure 1). Additionally, PHB’s interactome may be cell specific. This could be the case of another major contributor to ALS and FTD, the transactive response DNA-binding protein 43 (TDP-43), which also recruits PHB2 in mitochondria, as observed in a proteomic screening of mice cortex lysates and confirmed by immunoprecipitation by Davis and colleagues. However, the authors could not find a direct effect on mitochondrial bioenergetics in overexpression or downregulation of TDP-43 in HEK cell cultures (Davis et al., 2018).

### Prohibitins in neurodegenerative diseases

PD is another neurodegenerative disease related with PHB. Prohibitins have been associated with neuroprotection in substantia nigra (SN) and ventral tegmental area (VTA). In healthy human and mice brains, PHB levels are higher at VTA than in SN, as measured by WB (1.5- and 2-fold increase respectively), qRT-PCR (3-fold increase in mice) and immunofluorescence (≈2-fold increase in human brain) (Dutta et al., 2018). Interestingly, both regions show decreased PHB protein expression in PD human brains. However, in PD induced (MPTP treated) mice brains, the number of dopaminergic neurons is more decreased in SN as compared with VTA (Dutta et al., 2018), which suggest that higher levels of PHB in VTA may protect against neurodegeneration in this area. Indeed, in the SN of MPTP-treated mice the PHB levels increase 2-folds after 3 days of treatment, but decrease after 7 days, suggesting that PHB upregulation upon mitochondrial stress may be an early mechanism of neuroprotection. Similarly, in a 6-hydroxidopamine (6-OH)-induced PD rat model, PHB expression is upregulated in SN, and more in particular, in dying dopaminergic neurons, where PHB interacts with the mitochondrial complex I NADH-ubiquinone oxidoreductase 30 kDa subunit (NDUFS3), suggesting a possible role of PHB in neuroprotection of dopaminergic cells against mitochondrial dysfunction (Park et al., 2010). However, the role of PHBs in neurodegeneration is still unclear. In another study performed in mice by Triplett and colleagues, the authors performed a 2D-electrophoresis proteomic analysis in Ser/Thr kinase PTEN-induced kinase 1 (PINK1) deficient mutants, a mitochondrial gene associated with early-onset PD. They observed an overall decrease of PHB expression in PINK1 mutants (0.464-fold) as compared with control animals (Triplett et al., 2015). This goes in line with the growing evidences that PHBs regulate PINK1-PRKN/Parkin-dependent mitophagy (Wei et al., 2017; Yan et al., 2020). PHBs help to stabilize PINK1 in the outer mitochondrial membrane, who recruits Parkin to ubiquitinate membrane proteins and causing membrane rupture. This exposes PHB2 from the inner mitochondrial membrane, which interacts with phagophores to initiate mitophagy. It remains unclear though how the inhibition of mitophagy by lack of PINK1 could lead to a decrease of PHB expression. This suggest that the mechanisms regulating PHB expression in brain may be different depending on the tissue or the molecular pathways that promotes it. For example, protein quality control in mitochondria is a key component of mitochondrial homeostasis, and defects in these pathways can cause mitochondrial malfunction and neurodegeneration. Indeed, deficiency of the mitochondrial protease HtrA2 has been implicated in PD and causes motor neuron degeneration in mice. Goo and colleagues demonstrated using immunohistochemistry and luminescence assays that HtrA2 directly interact with PHB, which is overexpressed in HtrA2^−/−^ mutants, causing higher ROS production (≈3-fold increase) and abnormal mitochondrial membrane potential (≈40% decrease) (Goo et al., 2014). As in ALS and FTD, the correct assembly of mitochondrial complex I has also been associated with proteins involved in HD and PD, such as transglutaminase 2 (TG2). Mutant mice lacking this protein are more vulnerable to neurodegeneration of nigrostriatal cells upon treatment with disruptors of mitochondrial complex I and II. In a proteomic analysis by Battaglia and colleagues, and confirmed by SDS–PAGE and WB, they found that TG2 directly interacts with PHB, participating in the regulation of the respiratory chain by generating post-translational modifications on the PHB complex which facilitates the assembly of mitochondrial complex I (Battaglia et al., 2007).

### Protective role of prohibitin in the nervous system

On the other hand, in mice neuroblastoma cells and primary cortical neurons, overexpression of PHB protects the nervous system against hypoxia and cellular stress by fully reducing cytochrome *c* release, possibly by stabilization of cardiolipin in mitochondria and regulation of the AAA-protease OMA1 (Korwitz et al., 2016; Anderson et al., 2020). In another study, also in primary cortical neurons, PHB interacts with NO under glucose and oxygen deprivation to mediate neuroprotection, promoting a 50% increase of cell viability, which is not observed under NO deprivation (Qu et al., 2020). On the contrary, intense exercise in mice decreases PHB expression in neurons of dentate gyrus (≈40% decrease), while a moderate exercise promotes it (≈25% increase), as observed in quantification of immunohistochemistry images (So et al., 2017). However, it is not clear if the decrease is caused by a hypoxic environment or other exercise-associated causes. Similarly, it is not clear whether the increase of PHB is caused by promotion of cell proliferation and migration promoted by moderate exercise or by local induction of PHB expression (So et al., 2017; Davis et al., 2018). This data further highlights the need of deeper knowledge in PHB, as depending on the mitochondrial parameter of the study and the cause of a mitochondrial disfunction, the up or down regulation of PHB can be either beneficial or detrimental (Figure 1).

The protective role of PHB in apoptosis and oxidative stress has position this molecule as a strong candidate as a therapeutic target for ND and ageing. For example, Guyot and colleagues reported that in rat primary neuron cultures and mice, a purine derivative drug targets PHB1 and PHB2 with a binding affinity (k_D_) of 9.50 × 10^−6^ ± 4.60 and 1.29 × 10^−6^ ± 1.16 M, respectively, to improve cognitive deficits in ageing by regulating apoptosis and ROS production, as well as transcription of factors involved in synaptic function, neuroplasticity, and inhibition of neuronal Tau phosphorylation. Additionally, they reported that this purine drug reduces ≈2-folds the IL-β expression as measured by analysis of fluorescent images, *via* interaction with PHB, participating in the inhibition of neuro-inflammation (Guyot et al., 2020). Inversely, the role of PHB1 as a mitochondrial protease inhibitor may cause a more deleterious effect in protein aggregation. This is the case of mutations in F-box protein 7 (FBXO7), which causes juvenile PD. Mutations in FBXO7 cause a more severe aggregation in mitochondria, leading to toxicity and increased mitophagy, which can be further aggravated by PHB1 (Zhou et al., 2015). Considering that neurodegenerative diseases such as PD and AD implicate the misfold and aggregation of proteins, among other factors, it would be of interest to study the effect of PHB in protein aggregation under those backgrounds.

Another age-related disease is hearing loss, where ROS production induces mitochondrial damage and decreases its function. This causes a reduced mitophagy that is associated with higher cellular damage at aged cochlea and increased hearing loss. Interestingly, PHB2 expression is reduced (2.5-fold) along other mitophagy factors such as PINK1, Parkin and TOMM20 in mice with age-related hearing loss, which suggest a possible role of this protein in aging (Yu et al., 2021). Similarly, in a proteomic study by Sinclair and colleagues, PC12 cell cultures were treated with Aβ_42_, which caused a reduction in PHB2 expression (−1.591-fold decrease), suggesting a possible role for PHB in AD (Sinclair et al., 2021).

The role of PHB in neurodegeneration must be taken not only from a therapeutic point of view, but also as secondary risk factor of drug treatments for other diseases. For example, the antidepressant paroxetine has been shown to cause a decrease of PHB expression (0.47-fold decrease) in neuronal and glial mouse cell cultures (McHugh et al., 2008). Similarly, in SH-SY5Y cell cultures expressing the human mu-opioid receptor, a long term exposure to morphine causes a reduction of PHB expression of 38.4% ± 6.4% (Mouledous et al., 2005). On the contrary, commonly encountered natural products, such as mycotoxins in contaminated foods or chemical toxins in pesticides, can also cause an alteration in PHB expression. Indeed, Ochratoxin A (OTA), a mycotoxin present in mold particles from contaminated food causes a reduction of viability in mouse hippocampal HT22 cells, and the upregulation (3.5-fold increase) of PHB (Yoon et al., 2009). This suggest that PHB upregulation could be used as a protective mechanism against toxic insult, just as observed in neurons of PD-induced models (Park et al., 2010; Dutta et al., 2018). In dopaminergic SH-SY5Y cells treated with MPP^+^, overexpression of PHB restores mitochondrial membrane potential (≈2-fold increase as compared to control treated samples), decreases ROS (≈2-fold decrease) and reduces cytochrome c release (≈−0.8-fold decrease) (Dutta et al., 2018; Wang et al., 2021b), while knockdown of PHB increases the toxic effect (≈1.6-fold) of 6-OH as measured by cell viability (Park et al., 2010). Rotenone, another neurotoxin involved in mitochondrial complex I disruption, have been also associated with a protective role of PHB in PC12 cell cultures and rat primary neurons. Indeed, upregulation of PHB causes a decrease of ROS production (≈40% decrease after 15 min of rotenone induction) and restore mitochondrial complex I activity (≈10% increase) under rotenone treatment (Zhou et al., 2012; Anderson et al., 2018). Considering the wide role of PHB in regulating mitochondrial homeostasis, further studies on their inducibility are necessary, as well as elucidating their molecular interactome to develop more precise and targeted therapeutic treatments.

However, it is important to not place PHB as only a neuronal therapeutic target. For example, in human brains diagnosed with Schizophrenia, the right dorsolateral white matter area shows a higher density of prohibitin-expressing oligodendrocytes (Bernstein et al., 2012). In mice, PHB has been associated with dysfunction of mitochondria in Schwann cells and demyelination. PHB1 and PHB2 play diverse roles during developmental myelination and myelin maintenance in the peripheral nervous system (Poitelon et al., 2015). Depletion of PHB1 in Schwann cells also reduces levels of PHB2 protein, in agreement with their interdependence for PHB protein complex formation. First, PHB1 depletion activates mTORC1 and c-Jun in Schwann cells, which participate in the demyelination process (Della-Flora Nunes et al., 2021a). Della-Flora and colleagues also proposed that PHB2 has extra-mitochondrial activities during development, which are necessary for proper radial sorting, while both PHB1 and PHB2 are required in mitochondria for long-term myelin maintenance (Della-Flora Nunes et al., 2021b).

In summary, despite the important advances in the understanding of PHB’s function in mammals, their regulatory pathway and how they protect mitochondria from stress are still poorly understood. This is partly due to the fact that many studies have followed different approaches to study protein expression, going from proteomic screening to WB or qRT-PCR, and in many times, lack of molecular mechanisms is striking. Although the information provided by these results is of interest to the scientific community, a systematic approach should be followed to evaluate whether expression levels of PHBs, especially in downregulation, is actually caused by changes in cell proliferation or neurodegeneration rather than by regulation of PHB expression. Furthermore, the study of PHB’s interactome would be crucial to understand their role in neurodegenerative diseases, specially in those where protein aggregation is a particular characteristic, and where it seems that PHB may play a protective role. More studies are necessary to evaluate PHB as a potential therapeutic target to avoid possible secondary effects.

## Prohibitins in invertebrates

### Drosophila

The study of Prohibitins in the nervous system of invertebrate models is less abundant, although interesting progress has been achieved over the past few decades. In flies, the mechanisms regulating mitochondrial aggregation may be similar to those observed in mammals. The overexpression of human wild type FBXO7 in dopaminergic neurons of *Drosophila* also leads to neuron degeneration and FBXO7 aggregation (Zhou et al., 2015). If these effects can be aggravated by PHB1 in human cell lines, it would be interesting to study how PHBs participate in protein aggregation in simpler animal models such as *Drosophila*. More advances have been achieved with regard to the expression of PHB2 and its role in metabolic pathways *via* mitochondrial homeostasis. CG15081/l(2)03706 is the human ortholog of PHB2 in flies, and contains a U12-intron that is recognised by the splicing machinery, which plays an important role in gene regulation and mRNA splicing. Mutations in the splicing factor U6atac leads to downregulation of PHB2 (−1.29-fold), which may lead to the downregulation of other metabolic genes, including enzymes, nucleases, cytochrome P450 or detoxification-related transferases causing larval lethality (Pessa et al., 2010). It remains unclear though, whether these changes in the genetic profile caused by PHB2 downregulation could be also observed in a tissue-specific manner.

### Caenorhabditis elegans

In *C. elegans*, the study of PHB-1 and PHB-2 has focused on their effect on longevity, which is dependent on their metabolic state or other genotypes (Artal-Sanz and Tavernarakis, 2010). Knockdown of each of the subunits shorten lifespan in these animals (17 and 18 days, respectively, versus 20 days in wild type), however, in genetic backgrounds with mutations in signalling growth factors, mitochondrial homeostasis or metabolic proteins, PHB depletion has an opposing effect, dramatically extending lifespan to even three times longer than in wild type (Artal-Sanz and Tavernarakis, 2009a; b;^2010^). Interestingly, neuronal knockdown of *cco-1*, a member of the electron transport chain, induces the UPR^mt^, increasing mitochondrial HSP-6 expression (10-fold increase) in the intestine and extending lifespan (23.8 mean lifespan) (Durieux et al., 2011). The UPR^mt^ is also involved in lifespan regulation upon PHBs depletion (Yoneda et al., 2004; Gatsi et al., 2014), opening the possibility that neuronal manipulations of PHB could also have a systemic effect. Similarly, neuronal expression of an aggregation-prone polyglutamine (PolyQ40) induces the UPR^mt^ in the intestine (≈1.33-fold increase) and affects whole-animal physiology (Berendzen et al., 2016). It is unknown whether PHBs functions cell-autonomously and if depletion of PHBs in neurons or specific tissues may have an impact in the lifespan of these animals or in the development of neurodegenerative diseases.

### Other invertebrates

In planarians, interesting results have been observed also for PHB2, although its particular role in neurodegeneration is less studied in these organisms. However, they may reveal important features of PHB in stem cells considering that cell therapies are emerging strategies for the treatment of ND. Indeed, Rossi and colleagues knocked-down the *Phb2* ortholog in planarians, *DjPhb2*, and observed a blockage of regenerative capabilities of the stem cells, which eventually lead to death, suggesting that DjPhb2 is also involved in cell cycle proliferation and mitochondrial morphogenesis (Rossi et al., 2014). A role of PHB in neurogenesis has also been suggested in the sea urchin, where an expression of *Lv-prohibitin* has been observed in tissues where neurons of the pyloric and anal sphincters originate, although a neuronal role of *Lv-prohibitin* in this organism still needs to be proven (Slota et al., 2019). In insects, PHB2 acts as a receptor to mediate the internalization of the dengue virus (Kuadkitkan et al., 2010). Similarly, the enterovirus 71 (EV71), involved in neurological diseases, uses PHBs located at the cell surface to penetrate into the neurons and mitochondrial PHBs for viral replication (Too et al., 2018). This is an interesting characteristic of PHBs that would also be important to study in viruses targeting the central nervous system and may open the possibility of PHBs as targets for drug delivery.

In summary, research in PHB’s with invertebrate models has further increased our knowledge about their role in mitochondrial stress and ageing. The good use of these models can be taken to promote future research lines to explore interesting characteristics of these molecules, such as drug delivery, cell proliferation, or to study in deep their molecular mechanism.

## Future perspectives

**FIGURE 2:**
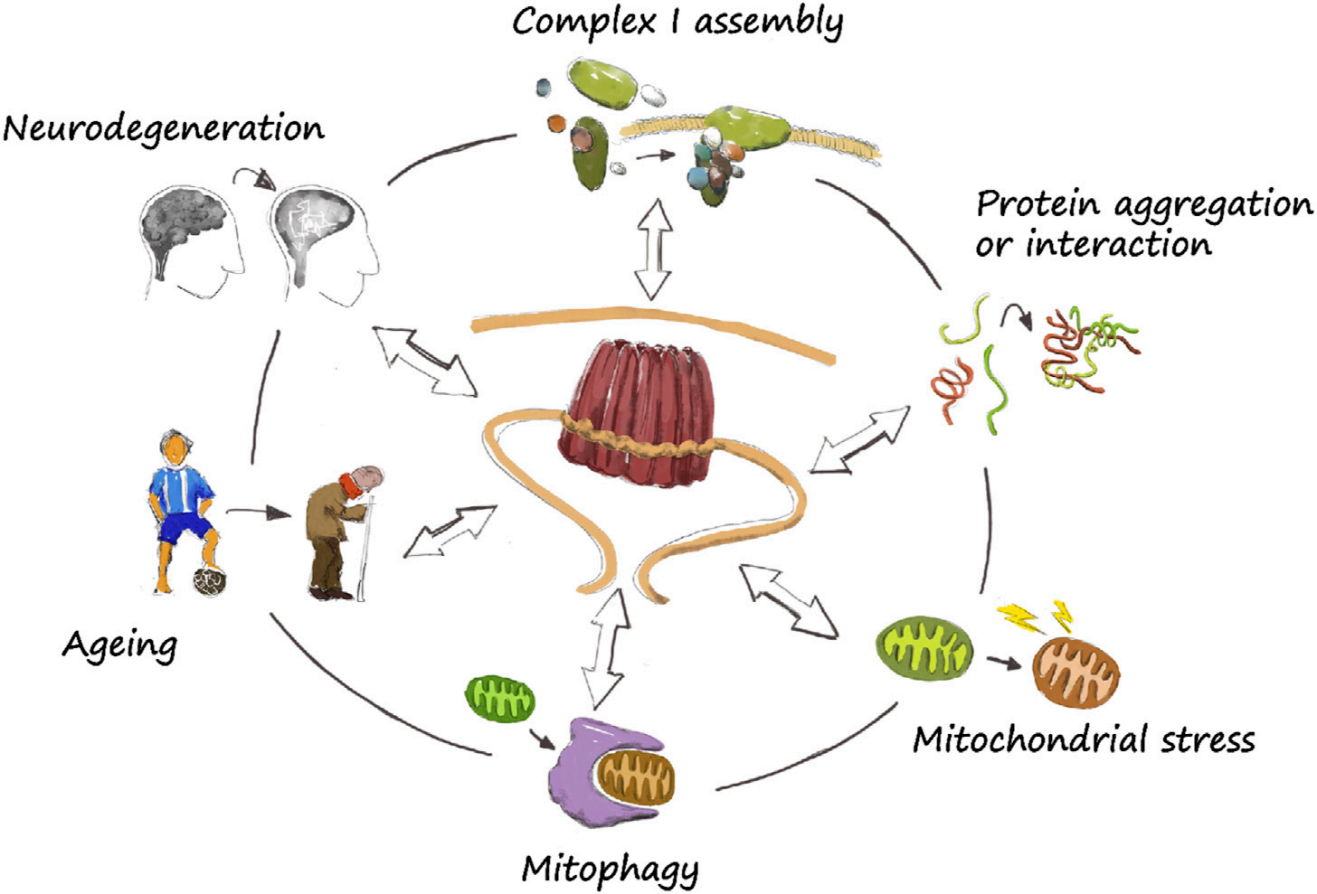
The prohibitin wheel. The role of PHBs in mitochondrial homeostasis is diverse. They directly participate in specific functions such as complex I assembly, protein aggregation and turnover, mitochondrial stress and mitophagy. They have been also described to have a role in ageing and neurodegeneration, although the exact mechanism by which they regulate lifespan or neuroprotection is still unclear. Nevertheless, a correct functioning of mitochondria is necessary, so a dysregulation of basic mitochondrial functions may lead to neurodegeneration.

The characterization of PHBs has enormously advanced in the recent years, and progress has been achieved in key points of its role upon mitochondrial homeostasis. Their biological function may be directly involved in specific roles, including complex I assembly, protein aggregation, induction of mitochondrial stress, and mitophagy, which are relevant in the ageing and neurodegenerative process (Figure 2). However, there is still much to learn about these proteins and its controversial role upon regulation of lifespan and neurodegeneration, although it seems to be an overall tendency to neuroprotection upon PHB upregulation (Table 1). Interestingly, a similar controversial characteristic is observed for PHB1 in liver injury and cancer, where it can be pro- or anti-tumorigenic, although the overall tendency is also protective [reviewed in Barbier-Torres and Lu (2020)]. It remains unclear though whether PHB2 has the same role in liver than his partner. Considering that neurodegenerative diseases are usually diagnosed at an advanced status, when symptoms become evident, PHB’s may stand as an early marker of diseases. Thus, a better understanding of the molecular mechanisms of PHBs in the regulation of mitochondrial homeostasis in ageing and neurodegeneration would be an important advance in the field (Figure 2). It remains unclear the global effect that neuronal PHBs may have in a whole organism or the central nervous system. Phb depletion has been shown to induce the UPR^mt^ (Gatsi et al., 2014; de la Cruz-Ruiz et al., 2021). Interestingly, mitochondrial stress in neurons induce the UPR^mt^ in other unrelated tissues, possibly mediated by neuropeptides (Durieux et al., 2011; Shao et al., 2016; Zhang et al., 2018). Advances in these important questions will be beneficial for human health and for the development of alternative therapeutic treatments.

**TABLE 1:** Summary of the studies involving PHB expression or function in the nervous system, ageing, and disease.

Subject	Related gene or disease	Treatment	Effect	Reference
Vertebrates				
Human	ALS and FTD	None	↓PHB1	Iridoy et al. (2018)
			↓PHB2	
Mice	*C9orf72* ^(−/−)^ (ALS and FTD)	None	Loss of PHB recruitment—mitochondrial complex I assembly	Wang et al. (2021a)
Human fibroblast/mice	CHCHD10^(−/−)^ (ALS and FTD)	None	SLP2 aggregation and dysregulation of motor neuron death	Genin et al. (2022)
Mice	TDP-43^(−/−)^ (ALS and FTD)	None	PHB2 recruitment in mitochondria	Davis et al. (2018)
Human	Healthy	None	PHBs levels in VTA > SN	Dutta et al. (2018)
	PD patients	None	↓ PHBs in VTA and SN	
Mice	PD-induced	MPTP	Degeneration in SN > VTA	
Rat	PD-induced	6-OH	↑PHBs in SN	Park et al. (2010)
		6-OH and Phb knockdown	↑Toxicity	
Mice	PINK1^(−/−)^ (PD)	None	↓ PHBs in brain	Triplett et al. (2015)
MEF	*HtrA2* ^(−/−)^ (PD)	None	↑ PHBs	Goo et al. (2014)
HEK cells	PD	*HtrA2* knock-down		
Mice	TG2^(−/−)^ (PD and HD)	None	TG2—PHB interaction to facilitate mitochondrial complex I assembly	Battaglia et al. (2007)
Mice neuroblastoma cells and primary cortical neurons	Healthy	*Phb* overexpression	Neuroprotection against hypoxia and cellular stress	Korwitz et al. (2016), Anderson et al. (2020)
Primary cortical neurons	Healthy	Glucose and oxygen deprivation	PHB interaction with NO—Neuroprotection	Qu et al. (2020)
Mice	Healthy	Intense exercise	↓PHB in neurons of dentate gyrus	So et al. (2017)
	Healthy	Moderate exercise	↑PHB in neurons of dentate gyrus	
Rat primary neurons	Aged	Purine derivative drug treatment	Interaction with PHB to improve cognitive deficits	Guyot et al. (2020)
Human fibroblast cells	*FBXO7* (PD)	None	Protein aggregation aggravated by PHB1	Zhou et al. (2015)
Mice	Healthy adult (Hearing loss)	None	↓PHB and ↓Mitophagy in cochlea	Yu et al. (2021)
Rat—PC12 cells	Healthy (AD)	Aβ_42_ treatment	↓PHB2	Sinclair et al. (2021)
Neuronal and glial mouse cell cultures	Healthy	Paroxetine	↓PHB	McHugh et al. (2008)
Neuroblastoma SH-SY5Y cells	Healthy	Morphine	↓PHB	Mouledous et al. (2005)
Mouse hippocampal HT22 cells	Healthy	Ochratoxin A	↓Viability	Yoon et al. (2009)
			↑PHB	
Dopaminergic SH-SY5Y cells	Healthy	*Phb* overexpression and MPP^+^ treatment	↑Neuroprotection	Wang et al. (2021b)
Rat—PC12 cells	Healthy	*Phb* overexpression and rotenone treatment	↑Neuroprotection	Zhou et al. (2012), Anderson et al. (2018)
Human	Schizophrenia	None	↑PHB in oligodendrocytes of right dorsolateral white matter	Bernstein et al. (2012)
Mouse NSC-34	Healthy	Enterovirus 71—infected	Cell surface PHB2 mediates virus internalization/Mitochondrial PHB2 facilitates virus replication	Too et al. (2018)
Invertebrates				
*Drosophila*	*U6atac* (Splicing factor)	None	↓PHB2	Pessa et al. (2010)
Planarian stem cells	Healthy	Knockdown of *Phb2*	Blockage of regenerative capabilities	Rossi et al. (2014)
Insects	Healthy	Dengue virus—infected	PHB2 mediates virus internalization	Kuadkitkan et al. (2010)
*C. elegans*	Healthy	*Phb* knockdown	Decreases lifespan	Artal-Sanz and Tavernarakis (2010)
		*Phb* knockdown + *daf-2* knockout	Increases lifespan	
